# The UK Research Excellence Framework and the Matthew effect: Insights from machine learning

**DOI:** 10.1371/journal.pone.0207919

**Published:** 2018-11-26

**Authors:** Lloyd D. Balbuena

**Affiliations:** Department of Psychiatry, University of Saskatchewan, Saskatoon, Canada; Max Planck Society, GERMANY

## Abstract

With the high cost of the research assessment exercises in the UK, many have called for simpler and less time-consuming alternatives. In this work, we gathered publicly available REF data, combined them with library-subscribed data, and used machine learning to examine whether the overall result of the Research Excellence Framework 2014 could be replicated. A Bayesian additive regression tree model predicting university grade point average (GPA) from an initial set of 18 candidate explanatory variables was developed. One hundred and nine universities were randomly divided into a training set (n = 79) and test set (n = 30). The model “learned” associations between GPA and the other variables in the training set and was made to predict the GPA of universities in the test set. GPA could be predicted from just three variables: the number of Web of Science documents, entry tariff, and percentage of students coming from state schools (r-squared = .88). Implications of this finding are discussed and proposals are given.

## Introduction

Many advantages of modern life are the fruits of scientific research and innovation. Yet in many countries, the proportion of research that is funded by the government has declined, while the share of industry has increased [1]. As a consequence, academic researchers are facing greater competition for funds. The competition among researchers and universities mirrors competition in the market [2], with the difference that success in the market is easy to measure (i.e. profit) and universally accepted. By contrast, research productivity has no universally accepted metric. For example, how does one rank an achievement in pure mathematics (e.g. proving Fermat’s last theorem) vis-a-vis an achievement in applied mathematics (e.g. the RSA algorithm, the basis of encryption in online banking)? This comparison requires a value judgment between knowledge that advances a discipline and knowledge that is practical [3]. Despite the difficulty of these comparisons, countries such as the UK, Australia, Italy and Germany engage in research assessment [4](and their scientists comply) because holding science accountable has been accepted as a social norm [5], and assessment exercises may be a “good enough” realization of a merit-based distribution of rewards [6].

Since 1986 and every six or so years thereafter, the UK has held research assessment exercises [7, 8]. The REF 2014 assessment evaluated three areas: research outputs (weighted at 65 percent), impact (20 percent), and environment (15 percent), all of which were rated by an expert panel [9]. The classification of each institution’s research quality across departments determined its grade point average (GPA). GPA multiplied by the number of units submitted for assessment became the basis for research funding allocation. Although the REF is intended to ensure that research funds are well-spent, the REF is a costly activity with estimates ranging from £47 million to £1 billion pounds [10, 11]. An official report to the UK’s funding bodies put the figure at £246 million—which equals the annual funding awarded to UCL (ranked second) and Cambridge (ranked third) for 2015–16 [10, 12]. Time-wise, each institution spent an average of 985 person-weeks [10]preparing submissions, an increase over the 2008 assessment [12]. These preparations included mock assessments in which academics were graded by panels of colleagues [4]. Those with unfavourable assessments faced the threat of job loss, denial of tenure, or transfer to a teaching only role [13]. These demands and challenges may have prompted the heads of the British Academy and the Royal Society to call for a less burdensome and costly alternative [14].

Several alternatives to the REF have been proposed [15, 16]. Generally, these alternatives rely on citation counts. The systematic analysis of citations, called bibliometrics, rests on two assumptions: first, that noteworthy scientific works elicit reactions from other scientists [17], and second, that citation count within the first year after publication predicts long-term scientific importance [18]. The h-index is the largest number of articles h that have been cited at least h times [19]. From this definition, it is clear that it has two components: article count and citation count, which represent productivity output and impact respectively [20]. Generally, these counts are not the same, but Hirsch’s definition constrains them to be equal, so it is the lower of the two counts that serves as the ceiling for the other. H-index is primarily calculated for each researcher but can be aggregated at the department or institution levels. With regard to the REF, h-index is proposed as an alternative (or complementary) metric for departmental research productivity and quality [15, 19, 21]. Recently, Mryglod and colleagues examined whether departmental h-indices predict departmental rank in REF 2014 [8]. Importantly, they published their predictions before the actual results were out. Later on their predictions were shown not to correspond with actual rank, and they concluded that h-index could not substitute for the REF [22]. While this might be the case, previous papers have found a correlation between citations and research assessment scores [23, 24]. Thus, citations and other factors could still play a role in research assessment.

As a starting point, it would be helpful to see the financial cost of the REF in light of what it intends to achieve: meritocracy and judicious spending. “Not spreading limited funds too thinly” is a condition for the UK to remain internationally competitive, according to a Russell Group report [25]. This view evokes the metaphor of competition, in which winners, who may have enjoyed advantages from the start, get rewarded—the so called Matthew effect. An alternative metaphor—one that is partly collaborative—can be given. The REF is a resource division exercise among several players, where the amount to be divided is reduced by how much the exercise itself costs. This implies that it would be in the interest of the players to minimize the administrative cost of the competition in order to have more money left over to divide. This is consistent with the objective of judicious spending. A cost-effective REF would serve the interests of peer reviewers, researchers, and all universities. Admittedly, universities that are weaker in research have more to gain compared to stronger ones, because the latter ones would take a large portion regardless of the size of the funding pie.

The present study had two objectives. First, to examine if a machine learning algorithm, applied to publicly available and library-subscribed data, predicts the REF 2014 GPA score. The REF itself used the following formula for overall GPA:

$$GPAScore=\frac{\left[\left(4\times pct\;of\;4^{*}scores)+(3\times pct\;{of\;3}^{*}scores)+(2\times pct\;of\;2^{*}scores)+(1\times pct\;{of\;1}^{*}scores\right)\right]}{100}$$

The starred numbers are categories ranging from 4* (world-leading) to 1* (recognised but modest), plus an extra “unclassified” category. Although these ratings ultimately determine rank, it can be argued that they in turn depend on universities’ accumulated human, social, and financial capital [26]and the extent to which institutions encourage or discourage research, as opposed to say, teaching [27]. These factors define the network or organizational context in which researchers are embedded [28]. A researcher’s affiliation opens or closes doors to resources, rewards, and the genesis and diffusion of ideas [29, 30], and these ultimately influence researcher productivity [27]. But research is not all about intangible factors. While the REF ostensibly rewards excellent *outputs*, it may actually reward amassed *inputs*. For example, while publishing in *Science* or *Nature* indicates a discovery of some importance [31], getting published there usually requires access to substantial funding. The housing and upkeep of laboratory mice might cost $200,000 annually [32]. Thus, the second objective of this project was to determine which among a wider set of factors most strongly predict REF performance. We required that these factors of research output be publicly available (or available through university libraries) in an attempt to reproduce the ranking at little or no cost.

## Materials and methods

Of the 128 institutions appearing in the REF 2014 ranking, 19 non-typical or specialized ones were excluded (e.g. Institute of Cancer Research, Open University, Cranfield University). This was done because many of the explanatory variables (described in the next section) are not applicable. One hundred and nine universities remained in our sample.

### Data sources and variables

The data for this study came from several different sources: the Times Higher Education report on REF 2014, HESA tables, Web of Science, and the Guardian league tables. Our selected predictor variables were of three kinds: institutional, faculty, and student characteristics. Institutional variables were: university income, total expenditures per student, number of full-time equivalent researchers submitted for assessment to the REF, and student-to staff ratio. Faculty variables were: citation impact (total citations / total papers), average h-index, percentage of faculty with a PhD, number of Web of science documents. Web of Science is a citation database offered on a subscription basis by Clarivate Analytics. It covers publications in the basic and life sciences, social sciences, and humanities. Bibliometric data were extracted for each university from the InCites database, restricted to the years 2008–13, corresponding to the period being evaluated. Student variables were: average entry tariff (a measure of how selective a university is by offering admission on the basis of high examination grades), percentage of socially disadvantaged students (defined as those coming from UK social classes 4 to 7), percentage of students from state schools, percentage of disabled students, percentage of students with ADHD, percentage of UK-domiciled students employed six months after graduation, average graduate salary, student satisfaction score from the National Student Survey, and career prospect score.

### Analysis

We used Bayesian additive regression trees (BART) [33] to predict GPA. The BART model “learns” associations between dependent variables and GPA in a subset of data and is then tested in a separate subset. This can be compared to a student who studies a set of topics and participates in a mock exam. The student might do well in it, but the basis for the grade is performance in the actual exam, which may or may not be similar to the mock exam. In like fashion, the BART model is “trained” using part of the data and is then assessed on its performance on the held-out data.

Conceptually, BART models are a type of regression model in which the dependent variable is predictable by a dichotomous split in one or more predictor variables, presented in sequence. For example, the best estimate for a car’s price might depend on the following nested questions. Is it imported? Is it a new model? Is its engine displacement greater than 1600 cc? By successively partitioning the sample according to the responses, an accurate estimate can be reached. Sketching a diagram of the series of questions would result in a tree-like structure with a number of terminal nodes, which represent answers to the questions. Creating several trees in like manner would result in trees of different structure and number of nodes. BART then creates a sum-of-trees model and performs regularization on the parameters of that model based on a set of priors that favour simplicity (i.e. fewer nodes) [33]. For technical details, kindly refer to the original BART paper [33].

**Fig 1:**
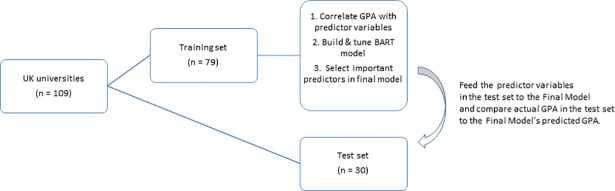
Schema of the analysis procedure.

The modelling process, consisting of the following steps, is depicted in Fig 1. First, we followed the 2/3 to 1/3 ratio recommendation for assigning units to a training and test set, with 79 universities assigned to a training set 30 to a test set [34]. The test set was set aside until step 4. In the training set, we calculated bivariate correlations of the 18 predictor variables plus GPA. Then, we fit a BART model with GPA as the dependent variable. Calibration and tuning were performed to determine hyperparameter values including: α and β (together with node depth, these give the probability that a node is nonterminal), *m* (number of trees), *k* (a parameter that controls how aggressive regularization is to be done), *q* (probability that BART has lower error than least squares regression), ν (a degrees of freedom to control the shape of residual error) [34, 35]. For this work, the hyperparameter values were: α = .95, β = 2, *m* = 20, *k* = 3, *q* = 0.95, ν = 3.

Finally, we examined which predictors were most relevant. BART’s algorithm builds trees—growing and pruning them iteratively to achieve better fit. In this process, only a small set of variables are used and BART keeps track of the frequency that each variable is used [33]. This serves as a measure of variable importance. We applied the final BART model to the test set. In this step, the model was given the values of predictors to be used for calculating fitted GPA. Predicted GPA was then compared to actual GPA and r-squared was calculated, which was used as an indicator of accuracy.

BART modelling was implemented in the R software [35] using the bartMachine package [36]. The data and syntax used in the analysis is available at: https://www.protocols.io/view/uk-ref-2014-analysis-data-and-r-script-t3eeqje.

## Results

Entry tariff (r = .78), percentage of faculty with doctoral degrees (r = .77), and h-index (r = .74) had the strongest correlations with GPA. (See Table 1). Inspecting which variables correlated with these three, we found that h-index was most strongly associated with university income, the number of full-time equivalent staff submitted to the REF for assessment, and the number of Web of Science documents for which the university was listed as an affiliation. Entry tariff was strongly inversely related to the percentage of students coming from state schools (r = -.89).

**Table 1:** Matrix of correlations in the training Set (n = 79).

		1	2	3	4	5	6	7	8	9	10	11	12	13	14	15	16	17	18	19
1	GPA	1.00																		
2	Percentage of overseas (non-EU) postgraduates	0.59	1.00																	
3	Citation impact	0.50	0.52	1.00																
4	h-index	**0.74**	0.46	0.65	1.00															
5	Mean graduate salary	0.51	0.54	0.36	0.63	1.00														
6	Average entry tariff	**0.78**	0.56	0.62	0.85	0.70	1.00													
7	Percentage of students with a disability	-0.32	-0.42	-0.22	-0.32	-0.54	-0.39	1.00												
8	Percentage of students with ADHD	-0.42	-0.45	-0.26	-0.44	-0.53	-0.43	0.84	1.00											
9	Percentage of graduates employed	0.27	0.08	0.07	0.29	0.12	0.38	-0.08	-0.06	1.00										
10	Percentage of students from classes 4 to 7*	-0.67	-0.48	-0.61	-0.75	-0.50	-0.83	0.14	0.18	-0.51	1.00									
11	Number of FTE staff assessed	0.66	0.44	0.57	**0.93**	0.59	0.81	-0.30	-0.38	0.26	-0.69	1.00								
12	Percentage faculty with PhDs	**0.77**	0.49	0.55	0.75	0.48	0.75	-0.23	-0.37	0.20	-0.68	0.66	1.00							
13	Student satisfaction	0.58	0.32	0.25	0.51	0.24	0.59	-0.15	-0.24	0.39	-0.57	0.44	0.58	1.00						
14	Expenditure per student	0.60	0.66	0.47	0.66	0.67	0.69	-0.45	-0.45	0.18	-0.54	0.66	0.50	0.32	1.00					
15	Student-to-staff ratio	-0.66	-0.40	-0.47	-0.75	-0.54	-0.75	0.30	0.38	-0.37	0.65	-0.74	-0.62	-0.48	-0.64	1.00				
16	Career prospects	0.69	0.48	0.38	0.69	0.74	0.81	-0.40	-0.42	0.48	-0.68	0.63	0.67	0.51	0.65	-0.70	1.00			
17	University income	0.63	0.42	0.57	**0.94**	0.62	0.78	-0.33	-0.43	0.21	-0.63	0.96	0.62	0.35	0.67	-0.70	0.62	1.00		
18	Students from State schools	-0.67	-0.52	-0.59	-0.84	-0.71	-**0.89**	0.29	0.29	-0.30	0.82	-0.83	-0.67	-0.49	-0.64	0.68	-0.73	-0.81	1.00	
19	Web of Science documents	0.60	0.39	0.55	**0.94**	0.61	0.78	-0.29	-0.38	0.23	-0.65	0.96	0.62	0.39	0.63	-0.72	0.62	**0.97**	-0.82	1.00

Notes: All p’s < .01. Figures appearing in bold font are discussed in the text. Social classes 4 to 7 include: small employers and own account workers, lower supervisory and technical occupations, semi-routine, and routine occupations.

**Fig 2:**
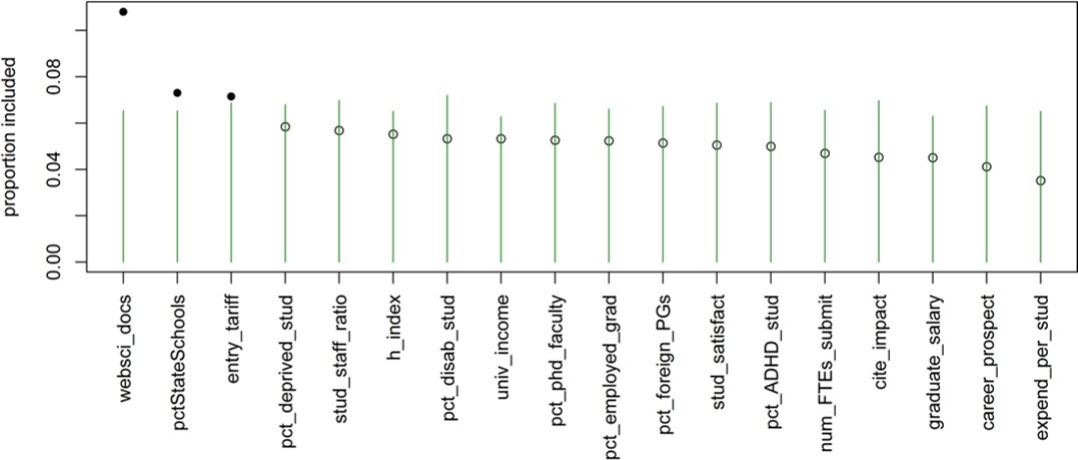
Variables ranked by order of predictive importance for GPA in the REF. Green lines indicate thresholds for inclusion into the model. Solid dot on top of green line indicates variable was selected as a significant predictor of GPA.

The BART model identified three of the 18 variables as important predictors (Fig 2). These were: number of Web of Science documents, entry tariff, and percentage of students coming from state schools. More Web of Science documents and higher entry tariff predicted higher GPA while percentage of state-school educated entrants was inversely related to GPA. (See Fig 2) The final model’s predicted GPA was strongly correlated with actual GPA of the test set (r = .94, r-squared = .88). The correlation and r-squared are based on the columns labelled Actual GPA and Predicted GPA in Table 2. However, the rank order of predicted GPA were discrepant with actual GPA. The largest discrepancies were with Roehampton University (Actual rank in the test set: 13^th^, Predicted rank in the test set: 23^rd^) and Central Lancashire University (Actual: 25^th^, Predicted: 18^th^). This error in ranking would have financial consequences in real-life: a £700,000 loss (20%) for Roehampton and a £240,000 gain (6%) for Central Lancashire, based on actual 2015 research funding allocations [37]. Interestingly, our model ranked all Russell universities in the test set at the top of the list together with University of St Andrews, as is the case in the actual ranking.

**Table 2:** Actual and predicted GPAs (Ranks) in the testing subset (n = 30).

Institution	Actual GPA	Predicted GPA	Actual Rank	Predicted Rank	Rank Discrepancy
Cambridge*	3.33	3.15	1	2	-1
York*	3.17	3.12	2	8	-6
Durham*	3.14	3.14	3	5	-2
St Andrews	3.13	3.15	4	2	2
Leeds*	3.13	3.13	4	7	-3
Newcastle*	3.09	3.21	6	1	5
Nottingham*	3.09	3.14	6	5	1
Birmingham*	3.07	3.15	8	2	6
Strathclyde	3.04	3.01	9	11	-2
Aberdeen	2.97	3.05	10	10	0
Leicester	2.93	3.08	11	9	2
Brighton	2.84	2.85	12	13	-1
Roehampton	2.83	2.59	13	23	-10
SOAS	2.82	3.00	14	12	2
Westminster	2.72	2.71	15	16	-1
West of England	2.70	2.78	16	15	1
Kingston	2.70	2.65	16	19	-3
Coventry	2.67	2.71	18	16	2
Glasgow Caledonian	2.67	2.62	18	20	-2
Oxford Brookes	2.66	2.82	20	14	6
Queen Margaret	2.65	2.38	21	27	-6
Middlesex	2.58	2.60	22	21	1
Lincoln	2.54	2.53	23	24	-1
Edinburgh Napier	2.52	2.60	24	21	3
Central Lancashire	2.51	2.69	25	18	7
London Met	2.44	2.45	26	26	0
Wales Trinity	2.39	2.37	27	28	-1
Anglia Ruskin	2.37	2.46	28	25	3
Bucks New	2.19	2.06	29	30	-1
Glyndwr	2.15	2.22	30	29	1

Asterisk (*) indicates Russell Group university

## Discussion

### General discussion about the results

The present study had two main findings. First, a machine learning algorithm applied to publicly available and library-subscribed data gave a good estimate of GPA. The second and more surprising finding is that Web of Science documents, entry tariff, and percentage of students coming from independent schools were the most valuable predictors of GPA. We now discuss the implications of these findings.

Web of Science documents measures quantity only and disregards quality or impact unlike the h-index. These two measures are related but do not correspond exactly. For example, the h-indices of deceased and retired scientists (who do not produce new papers) can only either increase or stay the same. On the other hand, a new scientist can publish 10 papers in one year but their h-index is 0 until their papers are cited. In a study that aimed to relate the h-index and 8 alternative indices with peer assessment, Bornmann and colleagues reported two important findings [20]. These indices consisted of two factors corresponding to impact and publication count. Compared with publication count, impact was more predictive of peer assessments. In the present work, publication count almost perfectly correlated with university income (r = .97) as did h-index (r = .94). These correlations do not indicate that income is a sufficient condition for publications and citations. They do confirm that scientific discovery and research excellence are subject to economic factors like everything else.

The strong correlation between Actual and Predicted GPA indicates that an inexpensive and time-saving technique could be used in conjunction with peer review. Using Cohen’s guide [38] for assessing strength of correlation, this would be a strong effect size (.26 or larger). Would it be reasonable to expect perfect prediction, i.e. for predicted GPA to equal actual GPA? We think that it is neither necessary nor desirable. There is an inherent trade-off between accuracy and cost in any evaluation process. Beyond a certain accuracy threshold there are diminishing returns to further improvement and the question whether £200 million is a fair cost (vis-à-vis a semi-automated REF) deserves serious discussion. A particularly wasteful use of time and money was the Canadian experience in which the cost of peer review exceeded giving every researcher a baseline grant [39]. Another source of measurement error is selection bias. To appreciate this point, it is worth pointing out that universities selected which staff (or units) it wanted to be assessed. For this reason, a rise in REF rank across time could simply be due to a more astute selection of staff instead of real progress [40]. Preparatory work for REF 2021 now includes a proviso to include all researchers.

That greater entry selectivity and fewer state-educated students should predict REF ranking at all highlight the tension between research excellence and social inclusion. It is to be expected that research-intensive universities rank higher in the REF. What the results suggest is that research excellence may partly result from a lack of diversity. Ultimately, universities are made up of people, so it is unsurprising that student and staff characteristics predict research output. Distinct processes that determine the composition of students and staff in UK universities are at work. First, elite UK universities select a high proportion of privileged students. In the years 2007–09, 18 percent of pupils from comprehensive schools were accepted into the Top 30 UK universities while the corresponding figure was 48 percent for independent schools [41]. Independent school pupils were nearly seven times as likely to be accepted to Oxbridge as those from comprehensive schools [41]. Second, privileged families select private schools and elite universities as a means of passing on wealth and status to their children. Recent research in the UK and US, suggest that wealthy families actively hinder downward mobility by ensuring that their children retain their social position even if they are of lower aptitude than lower class counterparts [42, 43]. Not surprisingly, the majority of Nobel scientists and Royal Society Fellows are the children of parents with professional and managerial-technical occupations [44, 45]. Third, children from underprivileged families (even with good grades) are less inclined to apply to elite universities because of financial and psychological barriers [46, 47].

A similar selection process is at work in universities. In a study of research productivity and prestige of academic position among biochemists, Long reported that departmental prestige has a stronger effect on productivity than prior publications [48]. This is interpreted as the accumulation of advantage that is responsible for the stratification of departments and universities into more and less prestigious groups [29]. This is manifested in the US by the stability of departmental prestige rankings from 1925 to 1993 despite the movement of people in and out of departments [29]. Consistent with the results of the present study, universities with the deepest pockets can also afford to hire the most reputable researchers [32].

The present study is subject to several limitations. First, typical universities (e.g. University of the Highlands and Islands, Leeds Beckett University) with a missing value in any of the 18 candidate predictors were excluded from our sample. This can be potentially remedied by requiring all universities to make their data publicly available. Second, as a proof of concept viability, we split UK universities into a training and testing set. As a result, our predictions of rank were limited to 30 universities. This would have to be modified in the real world application because all 120 or so universities would have to be part of the test set. One possibility in the next UK assessments cycle is to conduct panel reviews and machine learning components in parallel. For the machine learning part, the algorithm can be trained using REF 2014 data and feed it new data for testing. The departmental rankings of the two components could then be compared. Third, our model gives university-level GPA but not GPA at the department level. Variability across departments in a given university definitely affected our GPA estimate since the citation and document counts across disciplines are not comparable. For example, the average impact factors of molecular biology journals were 8 times greater than the average impact factors of mathematics journals [49]. Lastly, the prediction model relied solely on the Web of Science database. It has been reported that a researcher’s h-index, publication count, and citation count may vary across Google Scholar, Scopus, and Web of Science [50, 51].

### Proposals based on the results

In light of the findings of the present study, the following proposals regarding national research assessment exercises are given:

*Proposal 1: Consider incorporating machine learning into research assessment by applying it to human developed metrics of research excellence*. The advantages of using machine learning over human assessors only are: reproducibility, transparency, objectivity and the inclusion of all university researchers. Huge amounts of data about research outputs can be interrogated for publications, datasets shared, patents, software programs contributed, and other intellectual products. These outputs could be linked to university researchers who can be mandated to use a universal identifier such as ORCID to link them with research outputs and their affiliations. This step alone would free researchers from documenting, cherry-picking, and perhaps window-dressing their research outputs, leaving more time to do research itself. The biggest challenge would be the selection of appropriate indicators or metrics of research excellence. Several discussions on this topic have been held including the Science and Technology Indicators Conference in Leiden Netherlands [52], which led to the publication of proceedings and the Leiden Manifesto [53]. These and other documents could serve as the basis for formulating standard research metrics. Once these metrics are developed, the ranking process can be left to an open-source algorithm whose result can then be verified. The introduction of machine learning could also have disadvantages. First, depending on the algorithm used, understanding how the ranking was produced could be challenging. This is especially true of neural networks and deep learning that produce accurate predictions without giving people an insight about how they work [54]. Without understanding how the rankings were reached, universities would not know how to improve their performance in the next cycle. Secondly, algorithms are not immune to bias, and therefore care has to be taken so that human biases about gender, race, and reputations do not make their way into machine learning-based judgments of research excellence.

**Fig 3:**
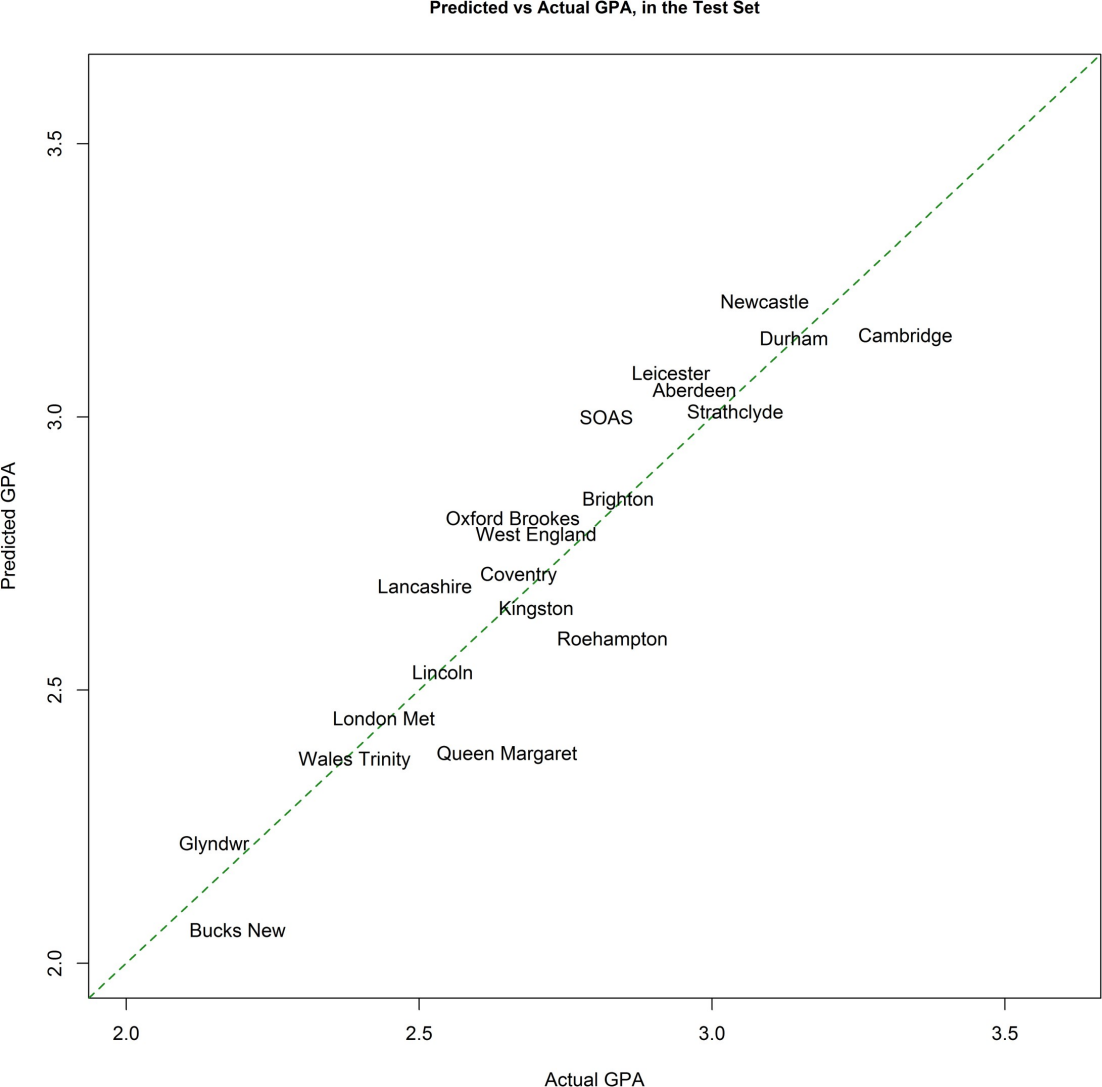
Predicted vs actual GPAs in the testing subset. Some universities are not displayed for clarity. See Table 2 for the complete list of universities in the test set with their actual and predicted GPAs.

*Proposal 2: Invest the savings from a partly automated REF exercise to fund research positions at universities lower-ranked in the REF*. The REF results are the basis for the allocation of quality-related (QR) funding. This amounts to about £12 billion for 6 years. Assuming that the use of machine learning could save 50 percent of the cost of panel-based research assessment, there would be *£125 million* available for strengthening research capacity in universities with less resources. The principal reason in support of this argument is that promoting excellence needs to be balanced with broadening the college of scientists [55]. The singular pursuit of excellence could result in “teaching only universities” that disseminate facts but not the spirit and methods of inquiry which are increasingly needed in a knowledge economy [55]. Accordingly, an independent review of the REF recommended that research excellence ought to be supported wherever it is found [56]. A second reason for redistributing potential REF savings is to mitigate the possibility of ranking error. This follows from the fact that 2 of the top 3 predictors of GPA are not research indicators as such (Fig 3). If the REF rankings were a race to the finish line, these student characteristics (as well as other advantages) may represent different starting points. A recent analysis showed that apart from Oxford and Cambridge, the rest of the Russell Group universities did not form a cluster of themselves only [57]. From a measurement perspective, this raises the question whether an ordinal ranking is possible, or whether clusters fit the data better. A third consideration for spreading research dollars is that research productivity, measured by number of publications, increases only up to a point [58]. For this reason, both the US National Institutes of Health and the Canadian Institutes of Health Research have decided to limit the size of grants any researcher can receive.

### Conclusion

Machine learning could be used alongside peer review in the UK’s research assessment exercises. The most important predictors represented factors not directly related to research impact. Nevertheless, the predicted ranking corresponded well with actual ranking and placed research-intensive universities at the top. This paradoxical result suggests that input factors in the form of financial and social capital play a role in research output. Incorporating machine learning into research assessment may reduce the burden of panel review. The savings in money and time could be reinvested in universities with lesser resources.
